# 974. The Use of Social Media for Medical Education During the COVID-19 Pandemic; A Vision to the Future

**DOI:** 10.1093/ofid/ofab466.1169

**Published:** 2021-12-04

**Authors:** Maria Jose Reyes Fentanes, Paula Amescua Guerra, Armelle Perez Cortes Villalobos

**Affiliations:** 1 Hospital Star Medica, Queretaro, Queretaro de Arteaga, Mexico; 2 Hospital ABC, Mexico City, Distrito Federal, Mexico; 3 UHN, Toronto, Ontario, Canada

## Abstract

**Background:**

The COVID-19 is the first pandemic in history where technology and social media can be used to keep people safe and informed. The correct management of information has been recognized as a critical part of controlling the COVID-19 pandemic. The objective of this study is to create a source of information about COVID-19 that is reliable, accessible, and easy to share while providing literature references.

**Methods:**

An Instagram account named @cienciacontracovid19 was created in 2020. In this account, the most relevant up-to-date medical information of COVID-19 is published daily in Spanish. All the account’s content is made by two infectious diseases specialists and a general practitioner. After 6 months since the creation of the account, we performed a survey to assess the followers perception of the usefulness of @cienciacontracovid19 during the pandemic.

**Results:**

The account was opened in November 2020. Figure 1 QR to access. Currently, the account has 9,534 followers from 5 Latin-American countries; 48% are between 25-34 years old, 76.6% are women, and 52% are healthcare workers. Until May 2021, 142 educational slides, 3 educational videos and 5 webinars have been posted. In the last 30 days, @cienciacontracovid19 has had 10,540 interactions and growth of +125% reaching 22,000 users. We conducted a survey in April 2021, in which 3,556 people answered. The following results were obtained: 76% considered that the information was always useful in their daily lives and 17% frequently useful. 77% affirmed that the information shared was always reliable and 47% consider that the information differed from other sources of information since it is easy to understand and 34% because it has bibliographic references to support it. 85% responded that the information shared in the account kept them from putting themselves at risk. When asking if the information shared has made them feel safer by being informed, 49% answered always and 44% frequently.

QR to access the instagram account

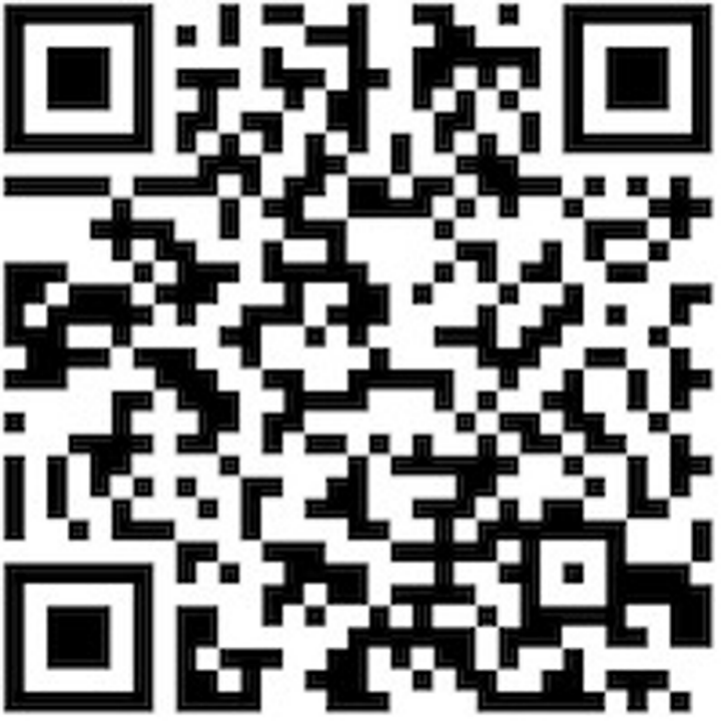

**Conclusion:**

@cienciacontracovid19 has been a valuable source of scientific information with a positive impact on its users. Its implementation has been a practical medical education tool during the COVID-19 pandemic. By being informed, people could potentially modify some of their behaviors to stay out of risk from COVID-19.

**Disclosures:**

**All Authors**: No reported disclosures

